# Network analysis of posttraumatic stress disorder and posttraumatic growth among patients with an intestinal stoma

**DOI:** 10.1016/j.apjon.2026.100988

**Published:** 2026-06-05

**Authors:** Lila Jiangenuer, Xiaomeng Wang, Wukelai Tuerdebieke, Subinuer Aihemaitiniyazi, Chen Liu, Zheng Xu, Xu Zhang

**Affiliations:** a School of Nursing, Peking University, Beijing, China; b Department of Surgery, The Second Affiliated Hospital of Liaoning University of Traditional Chinese Medicine, Shenyang, China; c Department of Gastrointestinal Surgery, Peking University First Hospital, Beijing, China; d School of Nursing, Sun Yat-sen University, Guangzhou, China

**Keywords:** Intestinal stoma, Posttraumatic stress disorder, Posttraumatic growth, Network analysis, Colorectal cancer

## Abstract

**Objective:**

Patients with colorectal cancer and an intestinal stoma often experience a complex psychological state where posttraumatic stress disorder (PTSD) coexists with posttraumatic growth (PTG). This study aimed to explore the interplay between PTSD and PTG symptoms in patients with an intestinal stoma to better understand their relationships.

**Methods:**

This cross-sectional study was conducted between January 2026 and March 2026, involving patients from three tertiary hospitals in Beijing, Liaoning, and Jilin provinces in China. Data were collected using a sociodemographic questionnaire, the PTG Inventory, and the PTSD Checklist for DSM-5. Network analysis was employed to identify central symptoms and potential bridge symptoms. Model stability and accuracy were assessed through bootstrap resampling.

**Results:**

Data from 416 participants were analyzed. The PTSD-PTG network identified the Posttraumatic Stress Disorder Checklist (PCL)10 ("Blaming yourself or someone else for the stressful experience or what happened after it") and the Posttraumatic Growth Inventory (PTGI) 5 ("A better understanding of spiritual matters") as the most central symptoms. Potential bridge symptoms linking PTSD and PTG included PCL20 ("Trouble falling or staying asleep") and PTGI1 ("My priorities about what is important in life"). The network model demonstrated acceptable stability and accuracy, although bridge expected influence estimates should be interpreted cautiously.

**Conclusions:**

This study identified potentially important PTSD and PTG symptoms and their interactions in patients with an intestinal stoma. Central and bridge symptoms may provide preliminary targets for clinical screening and individualized interventions to support both trauma-related distress and psychological growth. Longitudinal studies are needed to confirm these findings.

## Introduction

Colorectal cancer (CRC) is one of the most common malignant tumors worldwide, with a high incidence and mortality rate. In 2022, approximately 1.92 million new CRC cases were diagnosed globally, accounting for 10.2% of all new cancer cases, while CRC-related deaths totaled 904,000, representing 9.3% of all cancer fatalities.^1^ Ostomy surgery is an important treatment approach for CRC, particularly in patients requiring bowel diversion or sphincter preservation.^2^ The presence of an ostomy often leads to altered body image, difficulties in managing excretion, and reduced quality of life.^3^, ^4^, ^5^ These stressors, combined with the trauma of cancer diagnosis and treatment, frequently result in psychological distress.^6^^,^^7^

Posttraumatic stress disorder (PTSD) is a common mental health condition triggered by traumatic events, characterized by intrusive memories, avoidance behaviors, emotional dysregulation, and hyperarousal.^8^ Among cancer populations, PTSD has been increasingly recognized as a significant psychosocial burden. Studies among psychologically distressed cancer populations have reported that approximately 20% of patients experience PTSD symptoms within months of diagnosis.^9^ Specifically, in CRC patients, previous studies have reported PTSD prevalence in CRC patients ranging from 11.2% to 32.3%.^10^^,^^11^ Sustained psychological distress related to PTSD has been associated with a variety of adverse health outcomes, including increased susceptibility to infections,^12^ comorbidity with chronic pain conditions,^13^ somatic symptoms,^14^ and dysregulation of systemic immune function.^15^ These consequences can significantly impair patients’ quality of life and negatively affect their overall prognosis. On the other hand, some patients undergoing ostomy surgery also experience positive psychological changes, termed posttraumatic growth (PTG), which encompasses improved relationships, personal strength, and spiritual development.^16^^,^^17^ Previous studies suggest that PTG is relatively common among CRC patients following cancer diagnosis and treatment.^18^ PTG is an important concept as it represents the positive psychological changes that occur in individuals after experiencing adversity, helping alleviate psychological distress and fostering a more optimistic perspective on life.^19^ Several reviews have highlighted that PTG is positively correlated with improved quality of life (QOL) in cancer survivors.^20^^,^^21^ However, findings from Marziliano et al.^22^ challenge the assumption that PTG increases simply with time since diagnosis, as no evidence supported a positive association between temporal distance from cancer and PTG. This suggests that PTG may not unfold purely as a natural process over time and, instead, may depend on modifiable factors, highlighting the potential value of targeted psychosocial interventions. For example, Wang et al.^18^ conducted a systematic review and found that PTG development can be influenced by factors such as emotional states, coping mechanisms, and social environment. Interventions targeting these components have been shown to foster PTG and positively influence survivors' psychological well-being.

While PTSD and PTG are often considered distinct, they can coexist and interact in complex ways. PTSD and PTG may co-occur because traumatic experiences can simultaneously elicit distress and trigger adaptive growth processes. While PTSD reflects the negative emotional and cognitive responses to trauma, PTG emerges through meaning-making, coping, and re-evaluation of life priorities. Therefore, distress and growth are not mutually exclusive but may exist concurrently within the same individual following a traumatic event. Previous research has demonstrated varying relationships between PTSD and PTG, including positive, negative, and even U-shaped associations, where moderate PTSD symptoms are linked to the highest levels of PTG.^11^^,^^22^^,^^23^ For example, Marziliano et al.^22^ found a small but positive correlation between PTSD and PTG (*r* = 0.08) among cancer patients, with stronger associations observed in those with advanced-stage cancer. Similarly, in breast cancer survivors, PTSD is commonly observed in the early stages after diagnosis, while PTG tends to emerge after treatment.^24^ These findings suggest that PTSD and PTG might be interconnected, with some symptoms promoting growth while others exacerbate distress. However, the majority of studies have relied on traditional statistical methods that focus on linear correlations or isolated effects, often oversimplifying the complex relationships between these symptoms.^25^^,^^26^ In the case of CRC patients with ostomies, research has primarily focused on PTSD or PTG separately, with limited exploration of the intricate relationship between these two psychological states, leaving a critical gap in the literature.^18^^,^^27^^,^^28^ As a result, these studies may overlook critical nuances in how PTSD and PTG coexist and affect patients.

To bridge this gap, this study employed network analysis, a novel method in psychosomatic research, to explore the coexistence patterns of PTSD and PTG in CRC patients after ostomy surgery.^25^ Unlike traditional methods, network analysis treats symptoms as interconnected nodes within a larger system, enabling the identification of co-occurrence patterns, the roles of specific symptoms, and how they interact with each other.^25^ To further examine potential differences related to stoma type, we also conducted subgroup analyses comparing patients with permanent versus temporary stomas. By conceptualizing symptoms as interconnected nodes, this approach provides a richer understanding of the complex relationships between PTSD and PTG symptoms and identifies critical nodes that may serve as potential intervention targets.^26^ Ultimately, the findings from this study aimed to provide a scientific foundation for the development of targeted interventions, enhancing the psychological well-being and overall quality of life for patients living with intestinal stomas. Based on previous literature and theoretical considerations, we proposed the following hypotheses: H1: PTSD and PTG symptoms are interconnected within a network structure among patients with an intestinal stoma. H2: Hyperarousal-related PTSD symptoms (e.g., vigilance and sleep disturbances) may function as relatively central nodes within the network, whereas PTG-related symptoms involving interpersonal relationships, spiritual change, and re-evaluation of life priorities may serve as potential bridge symptoms linking distress and growth-related processes.

## Methods

### Study design and participants

This study employed a cross-sectional survey design. Participants were recruited from surgical wards of tertiary hospitals located in Beijing, Liaoning, and Jilin provinces between January 2026 and March 2026. Eligible participants were identified by clinical nurses based on the inclusion and exclusion criteria, and were then approached by trained research assistants who explained the purpose of the study. Patients who expressed interest were provided with written informed consent and, upon agreement, were enrolled in the study and asked to complete the survey. During the recruitment period, 606 patients were approached, of whom 437 agreed to participate and returned questionnaires. After screening for completeness of the PCL-5 and PTGI items, 416 questionnaires were deemed valid for analysis. The sample comprised 57.69% males and 42.31% females, with 24.04% of participants aged 60 years or above. In terms of stoma care levels, 19% of participants were completely or mostly dependent on others, while 81% were partially or fully self-sufficient.

We used the R package powerly to estimate the required sample size for our network model. The power analysis was conducted using Monte Carlo simulations following the method proposed by Constantin et al.^29^ A Gaussian graphical model with 41 nodes and an assumed edge density of 0.4 was specified. The target was to achieve a sensitivity of 0.6 with a probability of 0.8 (statistical power = 0.8). These parameter settings are commonly used in simulation-based power analyses of psychological network models. Using Monte Carlo simulations in *powerly*, testing sample sizes between 300 and 700 with 40 replications each, the recommended sample size was 352. This means that with 352 participants, we have an 80% chance to correctly identify 60% of the true edges in the network. We further validated this sample size using 3000 replications, confirming the reliability of this estimate. All data were prospectively collected for this study, including self-reported questionnaires and clinical records.

Eligible participants met the following inclusion criteria: (1) Adult patients (≥ 18 years) diagnosed with CRC who had undergone ostomy surgery and were fully aware of their diagnosis. (2) A minimum primary school education level, ensuring the ability to comprehend and complete the survey. (3) No significant cognitive or language impairments. Cognitive or language impairments were screened by reviewing medical records and conducting a brief orientation interview. Participants with diagnosed impairments or observable difficulties understanding study instructions were excluded. Participants were excluded if they met any of the following criteria: (1) Participants with significant psychiatric conditions were excluded from this study. To determine this, we reviewed each participant's medical records for documented diagnoses of major psychiatric disorders, such as schizophrenia, bipolar disorder, or severe depression. Additionally, participants were directly asked whether they had ever been diagnosed with such conditions by a health care professional. Those who reported or had records indicating significant psychiatric illness were excluded to ensure the reliability of self-reported data and the safety of participants. (2) Participants with severe physical illnesses or other malignant tumors. (3) Recent use of antidepressants or sedative medications, as these drugs may significantly influence psychological symptoms and confound the assessment of PTSD and PTG specifically related to the experience of living with a stoma. Inclusion of such participants could introduce bias due to medication-related effects on mood and cognition. (4) Experience of major family upheavals or traumatic events within the past six months, in order to minimize the influence of unrelated recent stressors on psychological outcomes. This exclusion criterion was designed to ensure that the PTSD and PTG measured were more likely attributable to the stoma-related experiences rather than other recent life events. (5) Participants concurrently enrolled in other clinical trials or research studies.

The study was conducted in accordance with the STROBE (STrengthening the Reporting of OBservational studies in Epidemiology) guidelines.

### Measures

#### General information survey

The General Information Survey was developed by the research team through a thorough literature review and consultation with nursing experts. It includes two main components: demographic information and disease-related information. Demographic data encompass details such as the participants' age, gender, and educational level. Disease-related information covers aspects like the stoma's location, the duration of stoma retention, the time elapsed since surgery, and the patient's self-care ability in managing the stoma.

#### Posttraumatic stress disorder symptom checklist

The Posttraumatic Stress Disorder Checklist (PCL) was originally developed by Weathers et al.^30^ based on the DSM-IV criteria and the PCL-5 was revised in 2013 to align with the DSM-5 symptom criteria. It is primarily used to assess the core symptoms of PTSD and their severity following exposure to a traumatic event. A validated Chinese version of the PCL-5 was used in this study and has demonstrated good reliability and validity in Chinese populations.^31^ The PCL-5 organizes PTSD symptoms into four clusters: recurrent traumatic experiences, persistent avoidance, negative alterations in cognition and mood, and persistently increased arousal. These clusters encompass a total of 20 symptoms. Each symptom is rated on a 5-point Likert scale, ranging from 0 ("not at all") to 4 ("extremely"), resulting in a maximum total score of 80. In this study, a total PCL-5 score of ≥ 33 was used as the clinical cutoff for PTSD screening. This threshold is consistent with prior validation studies^32^ suggesting that cutoff scores in the range of 31–33 provide optimal sensitivity and specificity for identifying probable PTSD cases. The McDonald's omega (ω) coefficients in this study were 0.970 (95% CI: 0.966 to 0.973).

#### Posttraumatic growth inventory

The Posttraumatic Growth Inventory (PTGI), developed by Tedeschi et al., in 1996,^33^ assesses positive psychological changes following traumatic events. A validated Chinese version of the PTGI was used in this study and has demonstrated good reliability and validity in Chinese populations.^34^ The scale consists of 21 items across five dimensions: (1) Relating to others; (2) New possibilities; (3) Personal Strength; (4) Spiritual Change; and (5) Appreciation of Life. Each item is rated on a six-point Likert scale from 0 to 5, with total scores ranging from 0 to 105. Higher scores indicate greater levels of PTG.^21^ The McDonald's omega (ω) coefficients in this study were 0.976 (95% CI: 0.973 to 0.979).

Both the PCL-5 and the PTGI have been widely used in cancer populations.^27^^,^^35^^,^^36^ For example, Zhou et al.^27^ conducted a network analysis of PTSD symptoms using the PCL-5 in a sample of 360 CRC patients undergoing chemotherapy in China.

#### Data collection

Prior to completing the questionnaire, all participants were required to sign an informed consent form. Trained members of the research team introduced the study's purpose, significance, and procedures to the participants using a standardized set of instructions, ensuring consistency across all sessions and minimizing potential biases. The questionnaires were administered in paper-and-pencil format and completed independently by patients with an intestinal stoma in a quiet, distraction-free environment. To ensure data quality, the completed questionnaires were collected on-site and immediately reviewed for any missing or incomplete responses. If omissions were identified, participants were asked to complete the missing information under the guidance of the research team. This real-time quality control process helped ensure data completeness and reduced the risk of missing values. Additionally, all data collection activities adhered to strict confidentiality protocols to protect participants' privacy and ensure compliance with ethical standards.

### Statistical analysis

The statistical analysis of this study involves both the sociodemographic characteristics of patients with an intestinal stoma and the partial correlation network analysis of PTSD and PTG symptoms. Sociodemographic and diagnostic data were processed and analyzed using SPSS 27.0 software. For categorical variables, frequencies and percentages were calculated, while continuous variables were presented as means and standard deviations.

For the partial correlation network analysis, R software (version 4.3.1) was used to construct and visualize the symptom network. The correlation matrix was computed using the cor_auto() function from the *qgraph* package, which automatically applies polychoric correlations for ordinal variables.^37^ Given the ordinal nature of the data and the widespread use of Gaussian graphical models in psychological network analysis, this approach was considered appropriate for estimating the symptom network. The *qgraph* package was employed to build a Gaussian graphical model (GGM), implemented through the graphical least absolute shrinkage and selection operator (glasso) algorithm. LASSO regularization was applied to eliminate spurious correlations and enhance the interpretability of the model. In this network, symptom nodes represent individual PTSD and PTG symptoms, while edges indicate partial correlations between nodes. Edge thickness corresponds to the strength of the correlation, and edge color represents its direction, with purple indicating positive correlations and red indicating negative correlations.

To identify core symptoms within the network, Expected Influence (EI) was calculated as the centrality measure, reflecting the relative importance of each symptom in maintaining the network structure. Additionally, Bridge Expected Influence (BEI) was computed to quantify the influence of symptoms that act as bridges between PTSD and PTG symptom clusters. A CS-coefficient of at least 0.25 is considered the minimum threshold for interpretability, while a coefficient greater than 0.50 is recommended to indicate acceptable stability.^38^ Therefore, BEI results with CS coefficients below 0.25 should be interpreted cautiously.

The stability of the network structure was assessed using bootstrap resampling techniques. Case-dropping bootstrapping was performed to test the stability of node centrality metrics. These procedures ensured that the network model was robust and that the findings were not influenced by sampling variability.

To examine potential differences between the symptom networks of participants with temporary and permanent ostomy, a Network Comparison Test was conducted using the NetworkComparisonTest package in R. This test evaluates differences in global network strength and overall network structure between groups through permutation testing.

## Results

### Sample

The characteristics of the study sample are summarized in Table 1. Percentages were calculated based on the total sample (*n* = 416) and reported to two decimal places.

**Table 1:** Sociodemographic and clinical characteristics of 416 patients with an intestinal stoma.

Variable	Category	Number of patients	Percentage (%)
Sex	Male	240	57.69
	Female	176	42.31
Age, years	< 40	37	8.89
	40–49	12	2.88
	50–59	267	64.18
	≥ 60	100	24.04
Marital status	Single	47	11.30
	Married	336	80.77
	Divorced or widowed	33	7.93
Monthly income (CNY)	< 1000	43	10.33
	1000–3000	121	29.09
	3001–5000	116	27.89
	5001–7000	74	17.79
	> 7000	62	14.90
Education level	Junior high school or below	151	36.30
	High school or technical secondary	144	34.62
	Bachelor's or associate degree	107	25.72
	Master's degree or above	14	3.37
Ostomy site	Ileal	239	57.50
	Colonic	177	42.50
Ostomy retention time	Permanent ostomy	205	49.30
	Temporary ostomy	211	50.70
Time since surgery	< 1 month	114	27.40
	1–3 months	130	31.25
	4–6 months	79	19.00
	6–12 months	12	2.88
	> 1 year	81	19.47
Ostomy care level	Completely dependent on others	39	9.38
	Mostly dependent on others	38	9.14
	Partial self-care (needs assistance)	188	45.19
	Fully self-sufficient	151	36.30

### PTSD status among patients with an intestinal stoma

The average total PTSD score (PCL-5) for the participants was 27.65 ± 14.96. Among them, 150 patients (36.1%) had PCL-5 scores at or above the clinical threshold of 33, indicating clinically relevant PTSD symptoms. The mean scores for the four PTSD dimensions were as follows: re-experiencing traumatic events (7.05 ± 3.75), persistent avoidance (2.77 ± 1.88), negative alterations in cognition and mood (9.91 ± 5.94), and increased arousal (7.92 ± 5.21). PTSD symptoms were observed in a substantial proportion of patients with an intestinal stoma, with varying severity across symptom dimensions. Further details are presented in Table 2.

**Table 2:** Total scores and dimensional scores of PTSD and PTG in patients with an intestinal stoma.

	Scoring range	Average (Mean)	Standard deviation (SD)
PTSD total score	0–80	27.65	14.96
Re-experiencing of traumatic experiences	0–20	7.05	3.75
Persistent avoidance	0–8	2.77	1.88
Negative changes in cognition and mood	0–28	9.91	5.94
Persistent increased arousal	0–24	7.92	5.21
PTG total score	0–105	42.64	19.51
Appreciation of life	0–15	13.31	6.09
Personal strength	0–20	6.52	3.15
New possibilities	0–25	8.45	4.04
Relating to others	0–35	6.37	3.21
Spiritual change	0–10	7.99	4.15

PTSD, posttraumatic stress disorder; PTG, posttraumatic growth.

### PTG status among patients with an intestinal stoma

The average total PTGI score was 42.64 ± 19.51, approximately corresponding to the midpoint of the 0–105 scale. The mean scores for the five PTG dimensions were as follows: Appreciation of Life (13.31 ± 6.09), Personal Strength (6.52 ± 3.15), New Possibilities (8.45 ± 4.04), Relating to Others (6.37 ± 3.21), and Spiritual Change (7.99 ± 4.15). While the scores varied across dimensions, these results suggest that patients generally reported experiences related to psychological growth following their illness and treatment experiences. Further details are presented in Table 2.

### Network structure and centrality indicators

**Fig. 1:**
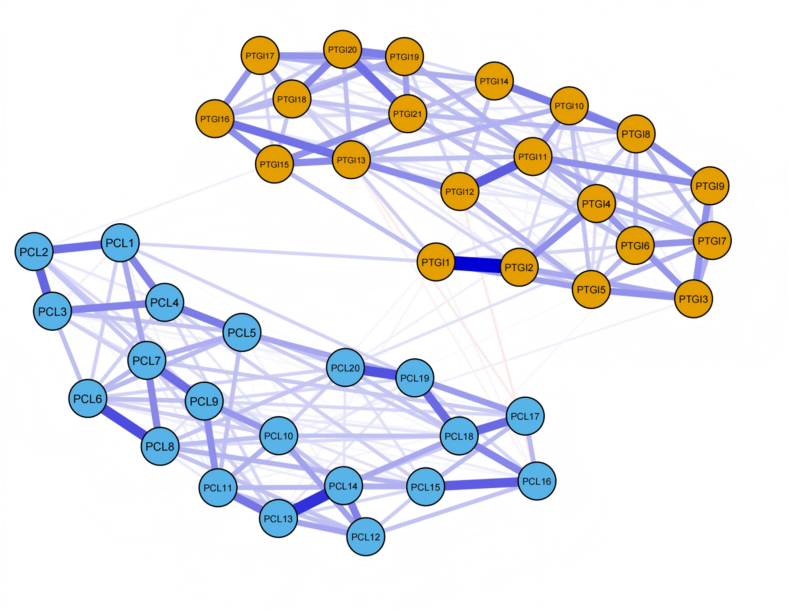
Network structure of PTSD and PTG symptoms in the full sample (*n* = 416). PTGI1: My priorities about what is important in life; PTGI2: An appreciation for the value of my own life; PTGI3: I developed new interests; PTGI4: A feeling of self-reliance; PTGI5: A better understanding of spiritual matters; PTGI6: Knowing that I can count on people in times of trouble; PTGI7: I established a new path for my life; PTGI8: A sense of closeness with others; PTGI9: Willingness to express my emotions; PTGI10: Knowing I can handle difficulties; PTGI11: I'm able to do better things with my life; PTGI12: Being able to accept the way things work out; PTGI13: Appreciating each day; PTGI14: New opportunities are available which wouldn't have been otherwise; PTGI15: Having compassion for others; PTGI16: Putting effort into my relationships; PTGI17: I'm more likely to try to change things which need changing; PTGI18: I have a stronger religious faith; PTGI19: I discovered that I'm stronger than I thought I was; PTGI20: I learned a great deal about how wonderful people are; PTGI21: I accept needing others. PCL1: Repeated, disturbing, and unwanted memories of the stressful experience; PCL2: Repeated, disturbing dreams of the stressful experience; PCL3: Suddenly feeling or acting as if the stressful experience were actually happening again; PCL4: Feeling very upset when something reminded you of the stressful experience; PCL5: Having strong physical reactions when something reminded you of the stressful experience; PCL6: Avoiding memories, thoughts, or feelings related to the stressful experience; PCL7: Avoiding external reminders of the stressful experience; PCL8: Trouble remembering important parts of the stressful experience; PCL9: Having strong negative beliefs about yourself, other people, or the world; PCL10: Blaming yourself or someone else for the stressful experience or what happened after it; PCL11: Having strong negative feelings such as fear, horror, anger, guilt, or shame; PCL12: Loss of interest in activities that you used to enjoy; PCL13: Feeling distant or cut off from other people; PCL14: Trouble experiencing positive feelings; PCL15: Irritable behavior, angry outbursts, or acting aggressively; PCL16: Taking too many risks or doing things that could cause you harm; PCL17: Being "superalert" or watchful or on guard; PCL18: Feeling jumpy or easily startled; PCL19: Having difficulty concentrating; PCL20: Trouble falling or staying asleep. PTSD, posttraumatic stress disorder; PTG, posttraumatic growth; PTGI, The Posttraumatic Growth Inventory; PCL, The Posttraumatic Stress Disorder Checklist.

**Fig. 2:**
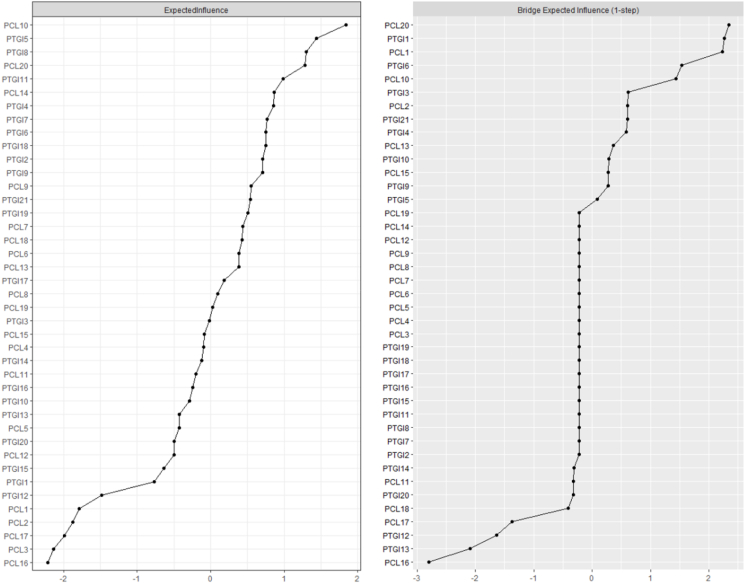
Symptom centrality estimates for the PTSD-PTG symptom network in the full sample (*n* = 416). PTSD, posttraumatic stress disorder; PTG, posttraumatic growth.

The PTSD and PTG symptom network for the overall sample of patients with an intestinal stoma is illustrated in Fig. 1, with centrality measures displayed in Fig. 2. The network analysis revealed several notable associations between symptoms. Among the edge weights, the strongest connections were observed between “Having gone through this experience, I have a better understanding of what is most important to me” (PTGI1) and “I have a greater appreciation for the value of my own life” (PTGI2) (weight = 0.38), as well as between “I felt distanced or isolated from other people” (PCL13) and “I found it difficult to experience positive emotions” (PCL14) (weight = 0.31).

For participants with a temporary ostomy, the strongest connections were observed between “Repeated, disturbing, and unwanted memories of the stressful experience” (PCL1) and “Repeated, disturbing dreams of the stressful experience” (PCL2) (weight = 0.20). The corresponding PTSD-PTG symptom network is presented in Supplementary Fig. S1, with centrality measures shown in Supplementary Fig. S2. For participants with permanent ostomy, the strongest connections were observed between “My priorities about what is important in life” (PTGI1) and “An appreciation for the value of my own life” (PTGI2) (weight = 0.34). The corresponding PTSD-PTG network symptom network is presented in Supplementary Fig. S3, with centrality measures shown in Supplementary Fig. S4.

The centrality analysis of the PTSD-PTG partial correlation network identified the most central symptoms based on standardized Expected Influence (EI) values. Specifically, for the overall sample, “Blaming yourself or someone else for the stressful experience or what happened after it” (PCL10, rEI = 1.84) and “Having a better understanding of spiritual matters” (PTGI5, rEI = 1.44) showed the highest centrality. These two symptoms demonstrated relatively higher influence within the estimated network structure. It is important to note that centrality here refers to statistical influence within the network and does not necessarily correspond to the physical position of nodes in the network visualization.

BEI analysis was conducted to explore potential bridge symptoms linking PTSD and PTG. Symptoms with relatively higher BEI values included “Difficulty falling or staying asleep” (PCL20, rBEI = 0.064), and “Having gone through this experience, I have a better understanding of what is most important to me” (PTGI1, rBEI = 0.062). However, these findings should be interpreted cautiously because the CS coefficient for BEI was below the recommended minimum threshold.

For participants with temporary ostomy, the symptom with the relatively highest EI was “Blaming yourself or someone else for the stressful experience or what happened after it” (PCL10, rEI = 1.59). The symptom with the relatively highest BEI value was “Knowing that I can count on people in times of trouble” (PTGI6, rBEI = 0.09) , as shown in Supplementary Fig. S5. For participants with permanent ostomy, the symptom with the highest EI was “Trouble experiencing positive feelings” (PCL14, rEI = 2.02). The symptom with the relatively highest BEI value was “My priorities about what is important in life” (PTGI1, rBEI = 0.12) , as shown in Supplementary Fig. S6.

Overall, PTSD and PTG symptoms were interconnected within the estimated network, and several symptoms demonstrated relatively higher estimates within the network.

### Stability analysis

The stability analysis of the partial correlation network in the full sample indicated a CS coefficient of 0.36 for EI, indicating moderate stability and exceeding the preset reference threshold of 0.25 (Supplementary Fig. S7). For BEI, the CS coefficient was 0.21 (Supplementary Fig. S8), indicating that bridge-related findings should be interpreted with caution due to limited stability. The stability of the network's edge weights was assessed using bootstrapping, with confidence intervals and significance plots presented in Supplementary Fig. S9. In the subgroup analyses, the CS coefficient for EI was 0.361 for participants with temporary ostomy and 0.36 for those with permanent ostomy, both exceeding the recommended threshold of 0.25 and indicating acceptable stability. Because the CS coefficient is a descriptive stability index derived from bootstrap resampling, formal statistical comparisons between CS values were not conducted.

### Network comparison

A Network Comparison Test was conducted to examine potential differences in network structures between participants with temporary and permanent ostomy. The results indicated that there was no significant difference in the overall network structure between the two groups (M = 0.203, *P* = 0.990). In addition, the global strength did not significantly differ between the temporary ostomy network (19.10) and the permanent ostomy network (18.80) (S = 0.298, *P* = 0.582). This suggests that the associations among PTSD symptoms and PTG were largely similar across the two groups.

The results were consistent with the primary complete-case analysis. Specifically, no significant difference in overall network structure was observed between participants with temporary and permanent ostomy (M = 0.203, *P* = 0.986). Similarly, the global strength did not significantly differ between the temporary ostomy network (19.10) and the permanent ostomy network (18.80) (S = 0.298, *P* = 0.569). These findings suggest that the main network results were generally consistent following multiple imputation.

## Discussion

### Main findings

To the best of our knowledge, this is one of the first studies to explore the symptom-level relationships between PTSD and PTG in people living with intestinal stomas using network analysis. By conceptualizing symptoms as interconnected rather than isolated entities, this study explored central and potential bridging symptoms within the PTSD-PTG network. The findings showed that “Blaming yourself or someone else for the stressful experience or what happened after it” (PCL10) and “A better understanding of spiritual matters” (PTGI5) were the most central symptoms in the overall network, while “Difficulty falling or staying asleep” (PCL20) and “My priorities about what is important in life” (PTGI1) emerged as potential bridge symptoms linking the two symptom clusters. These results provide a nuanced perspective on the co-occurrence patterns of distress and growth in this population. These findings partially supported our hypotheses, indicating that PTSD and PTG symptoms are interconnected and that certain symptoms play central or bridging roles within the network.

The findings revealed that “Blaming yourself or someone else for the stressful experience or what happened after it” (PCL10) emerged as the most central symptom in the PTSD-PTG partial correlation network. Self-blame, deeply rooted in the patients’ perception of dependency, reflects a critical psychological burden. Most patients in this study relied on assistance for daily living, and this loss of independence may be linked to feelings of inadequacy and guilt. The physical challenges associated with enterostomy, including impaired defecation control and complications such as infections or dermatitis,^39^ exacerbate this burden. These issues, together with the uncertainty and long-term discomfort related to CRC and its treatment, are commonly accompanied by negative emotions such as anxiety, depression, and reduced self-esteem.^5^ These psychological states may co-occur with self-blame and contribute to emotional distress.^40^ Addressing these emotional patterns may require not only medical care but also comprehensive psychological support.^41^

In contrast, “Having a better understanding of spiritual matters” (PTGI5) emerged as a key node representing growth and transformation. This finding aligns with prior research highlighting spiritual growth as a pivotal dimension of PTG.^33^ For many patients, the trajectory from diagnosis to treatment is often accompanied by profound reflection and a reevaluation of life's meaning and priorities. Although initially perceived as a misfortune, the experience of living with an enterostomy has been reported by some individuals to be associated with increased resilience, acceptance, and a renewed sense of purpose.^42^ These patterns may reflect a shift in perspective, whereby patients reinterpret their condition in broader existential or relational terms. Such findings suggest the potential importance of addressing patients' existential and psychological experiences during clinical care.^43^

Potential bridging symptoms between PTSD and PTG, such as “Trouble falling or staying asleep” (PCL20) and “My priorities about what is important in life” (PTGI1), may reflect potential points of interaction between distress and growth-related processes. Sleep disturbances often represent a physiological manifestation of PTSD,^44^ and may be influenced by ongoing physiological and psychological adjustments after ostomy surgery. In contrast, PTGI1 involves the re-evaluation of life priorities, a cognitive shift often associated with PTG.^45^ The presence of these symptoms as bridges may suggest that individuals who experience both distress and growth could engage in processes where affective dysregulation and cognitive restructuring coexist. While causality cannot be inferred from our cross-sectional data, these findings raise the possibility that targeting bridge symptoms—for instance, through sleep-focused interventions or values clarification techniques—may represent potentially clinically relevant points of interaction between PTSD and PTG processes. Similar patterns have been observed in other clinical populations. For instance, a study by Xu et al.^46^ on adult patients with systemic lupus erythematosus (SLE) in China found that poor sleep quality was negatively associated with PTG, while coping mechanisms like confrontation and social support were positively correlated with higher PTG scores. These results emphasize the potential role of sleep and coping strategies in post-trauma psychological adaptation. In this light, bridging symptoms like sleep disturbance may represent points of interaction between distress and growth-related mechanisms, warranting further investigation in longitudinal and intervention-focused studies. However, because the CS coefficient for BEI was relatively modest, the identification of bridge symptoms should be interpreted cautiously and considered exploratory.

Moreover, additional symptom connections—for instance, between PTGI1 (My priorities about what is important in life) and PCL1 (Repeated, disturbing, and unwanted memories of the stressful experience)—warrant further exploration. The interplay between cognitive restructuring and intrusive memories suggests that as individuals reflect on their life priorities (PTGI1), they may process distressing memories (PCL1) in a way that helps reframe their sense of meaning. For example, Menger et al.^47^ demonstrated how intrusive rumination in head and neck cancer survivors can evolve from distressing thoughts to more reflective meaning-making, a process that may be associated with PTG. Similarly, PTGI6 (Knowing that I can count on people in times of trouble), which reflects the role of social support, is linked with PCL20 (Trouble falling or staying asleep), a common PTSD symptom. The connection between these symptoms may suggest that strong social support (PTGI6) may interact with the emotional toll of sleep disturbances (PCL20). Interestingly, some conceptually related nodes—such as PTGI6 (“Knowing I can count on people”) and PCL13 (“Feeling distant or cut off from others”)—did not show a direct edge in the network. Although these constructs both reflect interpersonal connectedness, the absence of a direct association may be explained by the regularization procedure used in network estimation, which shrinks weaker associations toward zero. It is also possible that the relationship between perceived social support and social detachment is indirectly mediated through other PTSD or PTG symptoms in the network. This finding highlights the complex and potentially indirect interplay between PTG and PTSD symptoms.

PCL16 showed a relatively negative expected influence in the network, suggesting that risk-taking or self-harming behaviors may be inversely associated with several PTSD/PTG symptoms. This exploratory finding requires cautious interpretation. Such behaviors could reflect efforts to escape or numb distress, which may interfere with the cognitive restructuring and emotional engagement often associated with growth processes. Negative coping styles—such as impulsivity, self-blame, or behavioral disengagement—have been widely associated with elevated PTSD symptoms and reduced PTG.^18^^,^^48^^,^^49^ These findings highlight the potential clinical relevance of identifying maladaptive coping behaviors, which may serve as barriers to psychological growth following trauma. Targeting these negative coping styles through tailored psychosocial interventions may help support more adaptive psychological adjustment. Given the cross-sectional nature of our study, causal or temporal relationships between PTSD and PTG symptoms cannot be inferred. Network analysis reflects statistical associations among symptoms rather than directional effects. Future longitudinal research is warranted to clarify the temporal relationships between coping styles, PTSD symptoms, and PTG, as well as to explore the effectiveness of intervention strategies aimed at modifying coping patterns to foster PTG.

The results of these analyses were consistent with those of the primary complete-case analysis, suggesting that the exclusion of participants with incomplete questionnaires was unlikely to introduce substantial bias or materially affect the study findings.

### Implications for nursing practice and research

This study provides preliminary insights into the psychological symptom patterns of CRC patients with stomas. The identification of PCL10 and PTGI5 as statistically central symptoms suggests that they are highly interconnected within the PTSD-PTG symptom network and may reflect core experiences shared by this population. These symptoms could serve as meaningful reference points when designing psychosocial support strategies. In addition, bridging symptoms such as PCL20 and reevaluation of life priorities PTGI1 may be useful indicators of the interplay between distress and growth. While causal inferences cannot be drawn from this cross-sectional design, the network approach helps highlight potentially influential symptoms that warrant attention in clinical screening and supportive care. These findings may help inform clinicians in tailoring psychological interventions to address both trauma-related distress and opportunities for positive psychological change.

### Limitations

This study has several limitations. First, although participants were recruited from multiple centers, they were exclusively Chinese, which may limit the generalizability of the findings to other regions or cultural contexts. Future studies should aim to include larger and more diverse populations, including rural and underserved areas, to validate these results. Second, convenience sampling was employed, which may affect the representativeness of the sample and limit the generalizability of the findings. PTSD symptoms were assessed using a self-report instrument (PCL-5) rather than structured clinical diagnostic interviews, which may limit the precision of symptom classification. In addition, this sampling strategy may introduce potential selection bias, which should be considered when interpreting the results. Future research using more rigorous probability-based sampling methods is needed to enhance external validity. Third, this study's sample size estimation relied on Monte Carlo simulations using the *powerly* package, which inherently involves randomness and depends on assumptions about the underlying network structure and performance targets. As a result, the recommended sample size may carry some uncertainty and might not generalize perfectly to all scenarios.

In addition, the CS coefficient for BEI was below the recommended threshold, indicating limited stability of bridge centrality estimates. Therefore, bridge symptoms should be interpreted cautiously. In addition, the subgroup network analyses for temporary and permanent ostomy were based on relatively smaller sample sizes, which may reduce the stability of subgroup-specific edge weights and centrality estimates. Therefore, subgroup findings should also be interpreted cautiously.

## Conclusions

This study explored symptom-level relationships within the PTSD-PTG network among CRC patients with intestinal stomas. PCL10 and PTGI5 showed relatively higher centrality within the network, whereas PCL20 and PTGI1 may be potential bridge symptoms, although bridge centrality should be interpreted cautiously given its limited stability. These findings provide preliminary insights into the coexistence of distress and growth in this population and may inform psychological screening and supportive care. Longitudinal studies are needed to clarify temporal relationships. The network approach provides preliminary insights into the coexistence of distress and growth in this population.

## CRediT authorship contribution statement

**Lila Jiangenuer:** Conceptualization, investigation, Data collection, Formal analysis, Validation, Writing - original Draft, Writing - review & editing. **Xiaomeng Wang:** Conceptualization, Writing - review & editing, Formal analysis, Methodology, Visualization. **Wukelai Tuerdebieke and Subinuer Aihemaitiniyazi:** Validation, Formal analysis, Data curation. **Chen Liu:** Investigation, Resources. **Zheng Xu:** Supervision, Project administration, Investigation, Resources. **Xu Zhang:** Conceptualization, Data curation, Formal analysis, Funding acquisition, Methodology, Project administration, Resources, Software, Validation, Visualization, Writing - original draft. All authors have read and approved the final manuscript.

## Ethics statement

This study was approved by the Ethics Committee of School of Nursing, Sun Yat-sen University (Approval No. L2026SYSU-HL-005). Before participant recruitment and data collection at each participating hospital, institutional and departmental permission had been obtained from the corresponding hospital and department. The study was conducted in accordance with the Declaration of Helsinki. All participants provided written informed consent before completing the survey.

## Data availability statement

The data that support the findings of this study are available from the corresponding author, XZ, upon reasonable request.

## Declaration of generative AI and AI-assisted technologies in the writing process

During the preparation of this work, the authors used ChatGPT to assist with language editing and to improve the clarity of academic writing. After using this tool, the authors reviewed and revised the content and take full responsibility for the final manuscript.

## Funding

This study was supported by the 10.13039/501100001809National Natural Science Foundation of China (Grant No. 72404014); the Guangdong Provincial Medical Research Fund (Grant No. A2026241). The funders had no role in considering the study design or in the collection, analysis, interpretation of data, writing of the report, or decision to submit the article for publication.

## Declaration of competing interest

The authors declare no conflict of interest.
