# Supplementary material for: Network analysis of posttraumatic stress disorder and posttraumatic growth among patients with an intestinal stoma
Source: Asia Pac J Oncol Nurs. 2026 Jun 5;13:100988. doi: 10.1016/j.apjon.2026.100988 (PMC13292245; doi:10.1016/j.apjon.2026.100988)
Supplement: Multimedia component 1 [file mmc1.docx]

**Supplementary Materials**

1. **Supplementary Figure S1** Network structure of PTSD and PTG symptoms in patients with a temporary ostomy.
2. **Supplementary Figure S2** Symptom centrality estimates for the PTSD–PTG symptom network in patients with a temporary ostomy.
3. **Supplementary Figure S3** Network structure of PTSD and PTG symptoms in patients with a permanent ostomy.
4. **Supplementary Figure S4** Symptom centrality estimates for the PTSD–PTG symptom network in patients with a permanent ostomy.
5. **Supplementary Figure S5** Symptom BEI estimates for the PTSD–PTG symptom network in patients with a temporary ostomy.
6. **Supplementary Figure S6** Symptom BEI estimates for the PTSD–PTG symptom network in patients with a permanent ostomy.
7. **Supplementary Figure S7** Stability of EI estimates for the PTSD–PTG symptom network in the full sample.
8. **Supplementary Figure S8** Stability of BEI estimates for the PTSD–PTG symptom network in the full sample.
9. **Supplementary Figure S9** Bootstrapped edge weight stability of the PTSD–PTG symptom network in the full sample.


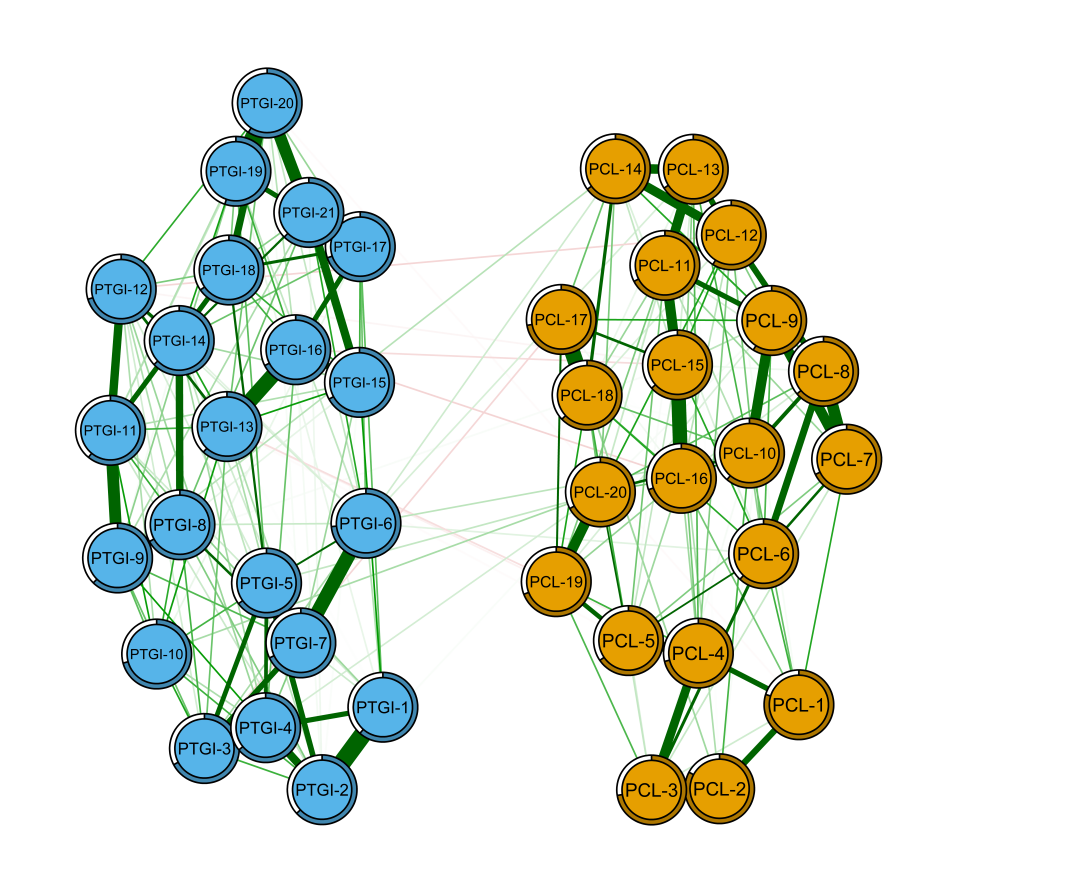


**Supplementary Figure S1** Network structure of PTSD and PTG symptoms in patients with a temporary ostomy.

**Note.** PTGI1: My priorities about what is important in life; PTGI2: An appreciation for the value of my own life; PTGI3:I developed new interests; PTGI4: A feeling of self-reliance; PTGI5: A better understanding of spiritual matters; PTGI6: Knowing that I can count on people in times of trouble; PTGI7:I established a new path for my life; PTGI8: A sense of closeness with others; PTGI9: Willingness to express my emotions; PTGI10: Knowing I can handle difficulties; PTGI11: I'm able to do better things with my life; PTGI12: Being able to accept the way things work out; PTGI13: Appreciating each day; PTGI14: New opportunities are available which wouldn't have been otherwise; PTGI15: Having compassion for others; PTGI16: Putting effort into my relationships; PTGI17: I'm more likely to try to change things which need changing; PTGI18: I have a stronger religious faith; PTGI19: I discovered that I'm stronger than I thought I was; PTGI20: I learned a great deal about how wonderful people are; PTGI21: I accept needing others. PCL1: Repeated, disturbing, and unwanted memories of the stressful experience; PCL2:Repeated, disturbing dreams of the stressful experience; PCL3: Suddenly feeling or acting as if the stressful experience were actually happening again; PCL4: Feeling very upset when something reminded you of the stressful experience; PCL5: Having strong physical reactions when something reminded you of the stressful experience; PCL6: Avoiding memories, thoughts, or feelings related to the stressful experience; PCL7: Avoiding external reminders of the stressful experience; PCL8: Trouble remembering important parts of the stressful experience; PCL9: Having strong negative beliefs about yourself, other people, or the world; PCL10: Blaming yourself or someone else for the stressful experience or what happened after it; PCL11: Having strong negative feelings such as fear, horror, anger, guilt, or shame; PCL12: Loss of interest in activities that you used to enjoy; PCL13: Feeling distant or cut off from other people; PCL14: Trouble experiencing positive feelings; PCL15: Irritable behavior, angry outbursts, or acting aggressively; PCL16: Taking too many risks or doing things that could cause you harm; PCL17: Being "superalert" or watchful or on guard; PCL18: Feeling jumpy or easily startled; PCL19: Having difficulty concentrating; PCL20: Trouble falling or staying asleep.





**Supplementary Figure S2** Symptom centrality estimates for the PTSD–PTG symptom network in patients with a temporary ostomy.


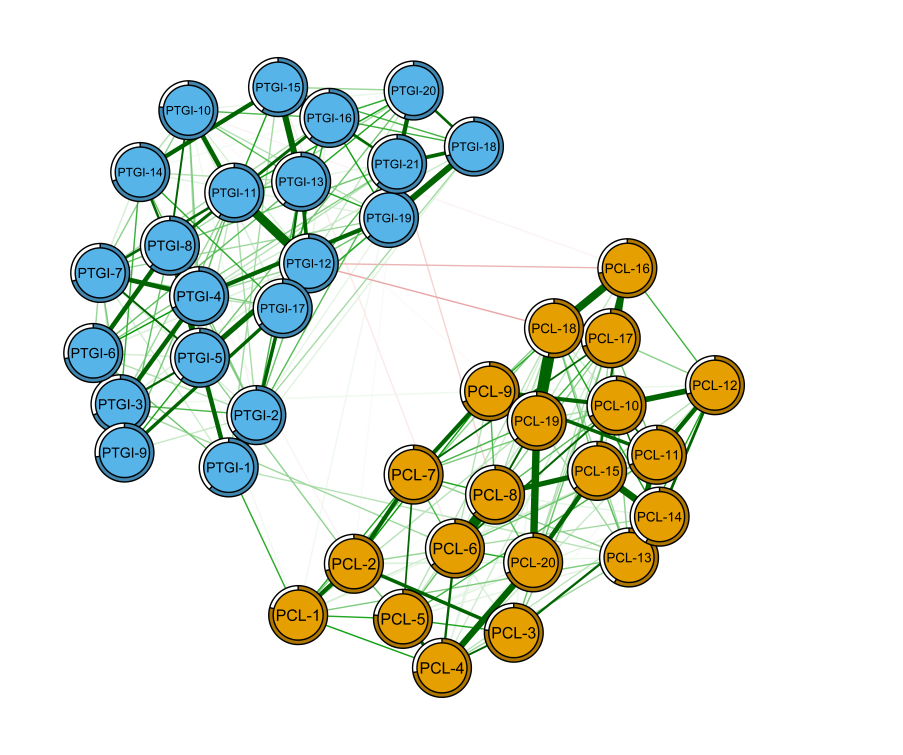


**Supplementary Figure S3** Network structure of PTSD and PTG symptoms in patients with a permanent ostomy.

**Note.** PTGI1: My priorities about what is important in life; PTGI2: An appreciation for the value of my own life; PTGI3:I developed new interests; PTGI4: A feeling of self-reliance; PTGI5: A better understanding of spiritual matters; PTGI6: Knowing that I can count on people in times of trouble; PTGI7:I established a new path for my life; PTGI8: A sense of closeness with others; PTGI9: Willingness to express my emotions; PTGI10: Knowing I can handle difficulties; PTGI11: I'm able to do better things with my life; PTGI12: Being able to accept the way things work out; PTGI13: Appreciating each day; PTGI14: New opportunities are available which wouldn't have been otherwise; PTGI15: Having compassion for others; PTGI16: Putting effort into my relationships; PTGI17: I'm more likely to try to change things which need changing; PTGI18: I have a stronger religious faith; PTGI19: I discovered that I'm stronger than I thought I was; PTGI20: I learned a great deal about how wonderful people are; PTGI21: I accept needing others. PCL1: Repeated, disturbing, and unwanted memories of the stressful experience; PCL2:Repeated, disturbing dreams of the stressful experience; PCL3: Suddenly feeling or acting as if the stressful experience were actually happening again; PCL4: Feeling very upset when something reminded you of the stressful experience; PCL5: Having strong physical reactions when something reminded you of the stressful experience; PCL6: Avoiding memories, thoughts, or feelings related to the stressful experience; PCL7: Avoiding external reminders of the stressful experience; PCL8: Trouble remembering important parts of the stressful experience; PCL9: Having strong negative beliefs about yourself, other people, or the world; PCL10: Blaming yourself or someone else for the stressful experience or what happened after it; PCL11: Having strong negative feelings such as fear, horror, anger, guilt, or shame; PCL12: Loss of interest in activities that you used to enjoy; PCL13: Feeling distant or cut off from other people; PCL14: Trouble experiencing positive feelings; PCL15: Irritable behavior, angry outbursts, or acting aggressively; PCL16: Taking too many risks or doing things that could cause you harm; PCL17: Being "superalert" or watchful or on guard; PCL18: Feeling jumpy or easily startled; PCL19: Having difficulty concentrating; PCL20: Trouble falling or staying asleep.





**Supplementary Figure S4** Symptom centrality estimates for the PTSD–PTG symptom network in patients with a permanent ostomy.





**Supplementary Figure S5** Symptom BEI estimates for the PTSD–PTG symptom network in patients with a temporary ostomy.





**Supplementary Figure S6** Symptom BEI estimates for the PTSD–PTG symptom network in patients with a permanent ostomy.


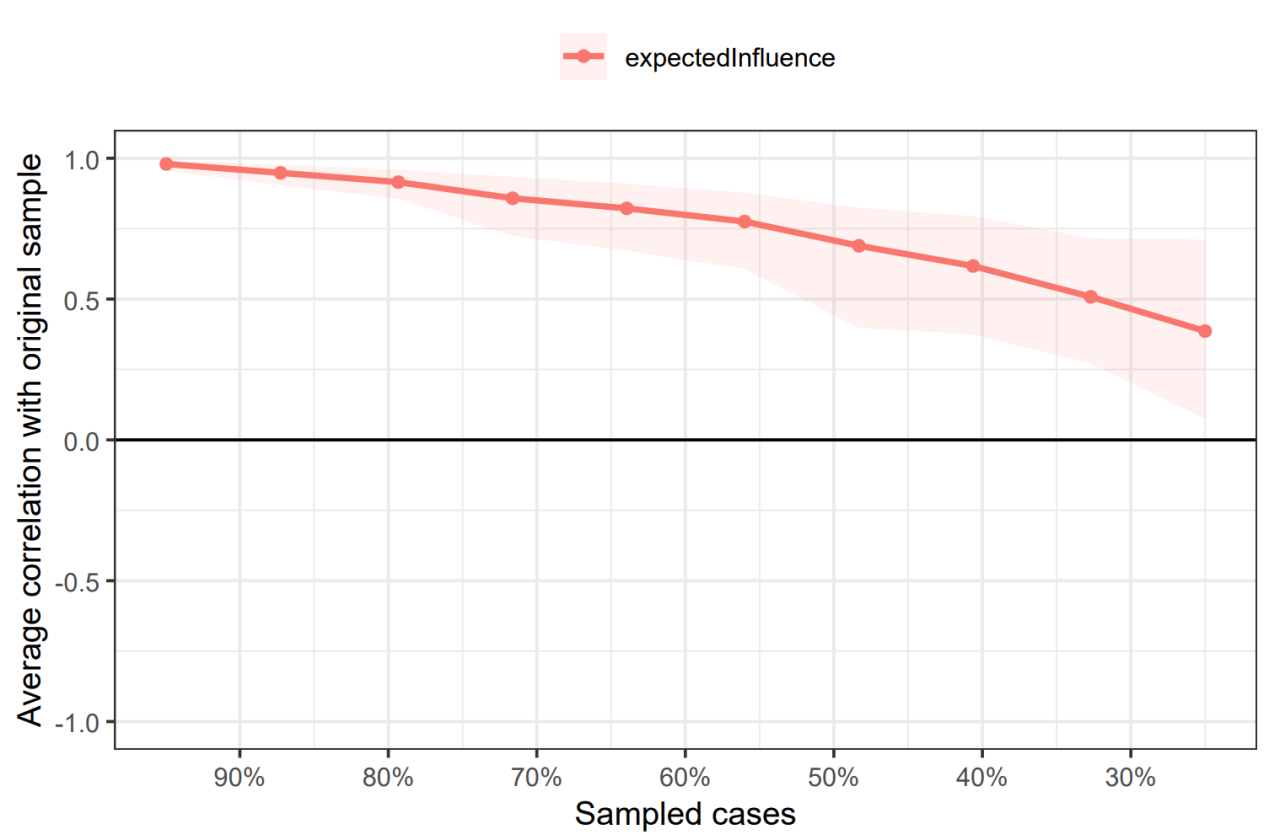


**Supplementary Figure S7** Stability of EI estimates for the PTSD–PTG symptom network in the full sample.


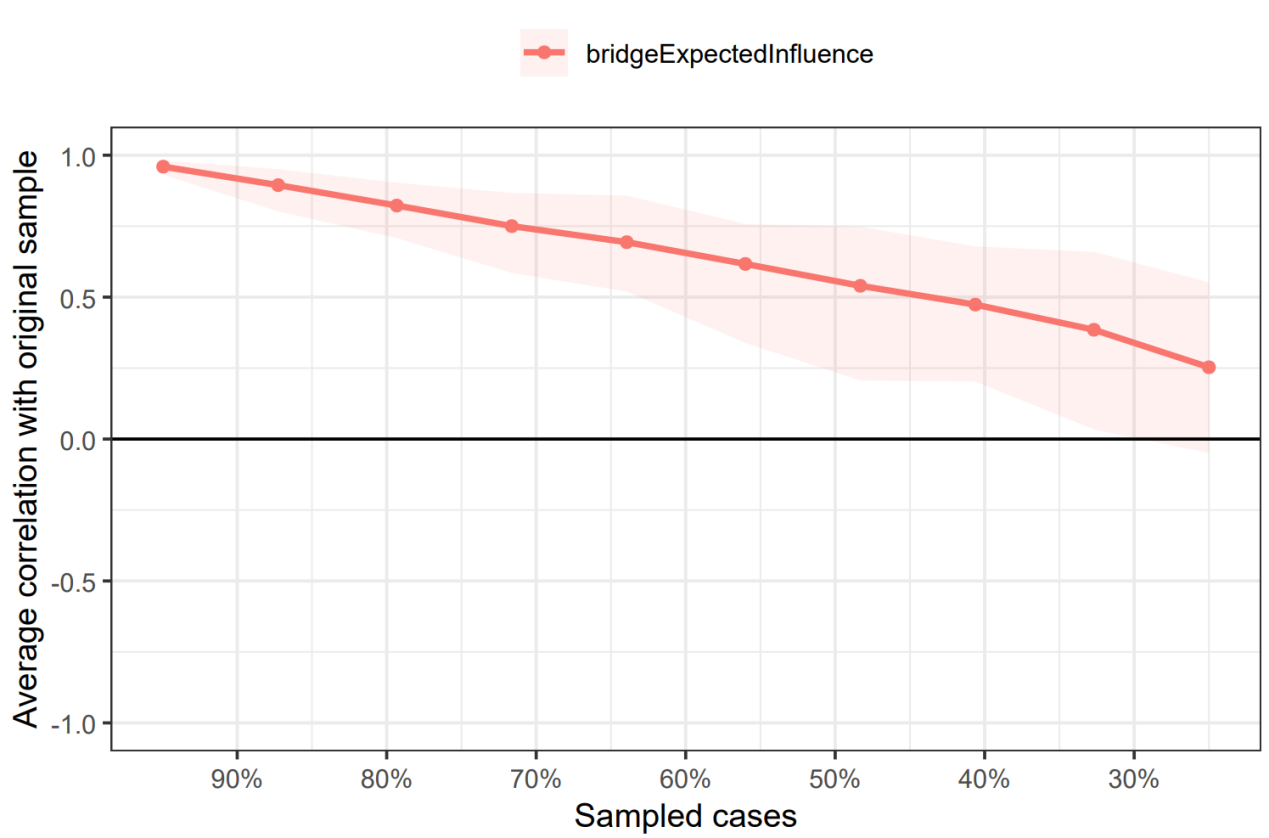


**Supplementary Figure S8** Stability of BEI estimates for the PTSD–PTG symptom network in the full sample.


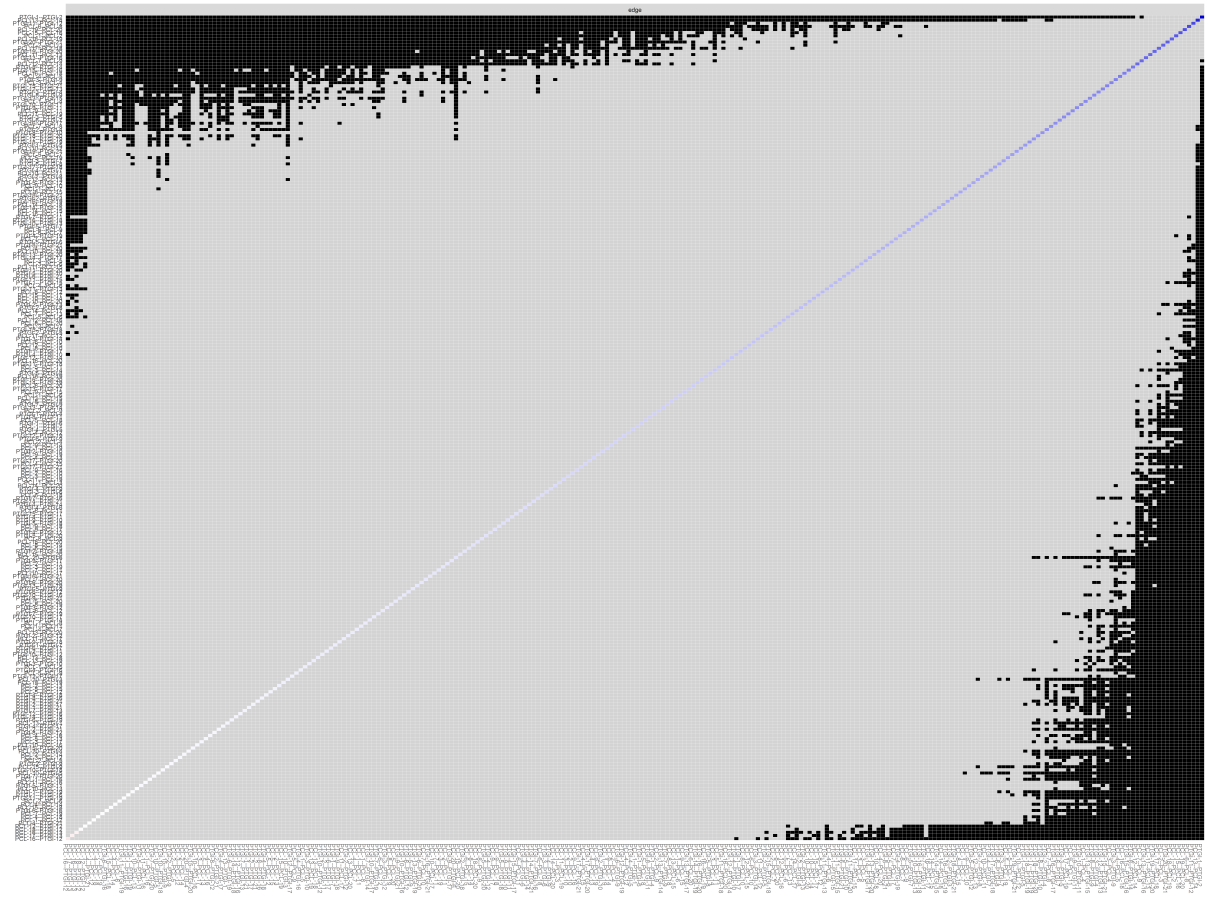


**Supplementary Figure S9** Bootstrapped edge weight stability of the PTSD–PTG symptom network in the full sample.
